# Rapid, Direct, Noninvasive Method to Determine the Amount of Immobilized Protein

**DOI:** 10.1021/acs.analchem.2c05402

**Published:** 2023-03-20

**Authors:** Rok Ambrožič, Rok Mravljak, Aleš Podgornik

**Affiliations:** † Faculty of Chemistry and Chemical Technology, University of Ljubljana, Večna Pot 113, 1000 Ljubljana, Slovenia; ‡ COBIK, Mirce 21, 5270 Ajdovščina, Slovenia

## Abstract

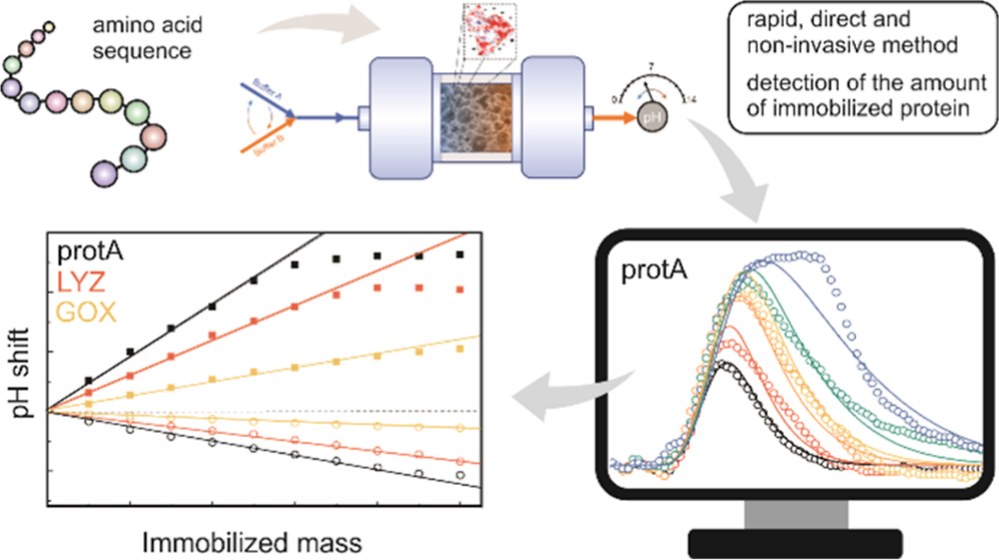

Protein immobilization is of utmost importance in many areas, where various proteins are used for selective detection of target compounds. Despite the importance given to determine the amount of immobilized protein, there is no simple method that allows direct, noninvasive detection. In this work, a method based on pH transition, occurring during change of solution ionic strength, was developed. The method utilized the ionic character of the immobilized protein while implementing biologically compatible buffers. Five different proteins, namely, glucose oxidase, horseradish peroxidase, bovine serum albumin, lysozyme, and protein A, were immobilized in different amounts on a porous polymeric matrix, and their pH transition was measured using lactate buffer of various concentrations and pH values. A linear correlation was found between the amount of immobilized protein and the amplitude of the pH transition, allowing the detection down to 2 nmol of immobilized protein. By changing the buffer concentration and pH, the sensitivity of the method could be tailored. Criteria based on the symmetry of the pH transition peak have been developed to determine if a particular measurement is within a linear range. In addition, a mathematical model was developed enabling prediction of pH transition profiles based solely on the protein amino acid sequence, the buffer p*K*_a_ value(s), and the amount of immobilized protein.Hence, it can be used to design pH transition method experiments to achieve the required sensitivity for a target sample. Since the proposed method is noninvasive, it can be routinely applied during optimization of the immobilization protocol, for quality control, and also as an in-process monitoring tool.

## Introduction

Protein immobilization is important in many different areas where detection of specific molecules or their conversion is required. Immobilized proteins can be either enzymes enabling selective conversion or affinity ligands for adsorption of specific molecules. A variety of different protein affinity ligands are immobilized on small volume devices such as enzyme-linked immunosorbent assays,^1^ microfluidic devices,^2−4^ and biosensors.^5−7^ On the other hand, there are many large-scale processes using immobilized proteins in the food, chemical, pharmaceutical, cosmetic, and medical device industries either for bioconversion^8^ or as selective ligands in downstream processing,^9^ for example, in the isolation of monoclonal antibodies.^10^ Depending on the immobilization support, protocol, and linking chemistry, the properties of the immobilized protein may change compared to the native protein.^11^ This may be due to structural changes in the protein itself, a different microenvironment, proximity of the matrix,^8,12^ or a high density of the immobilized protein.^13^ For immobilized enzymes, this leads to a change in their catalytic properties such as the reaction kinetics, the specificity, and a shift in the pH optimum,^12^ while for affinity ligands, this usually translates into lower binding efficiency.^14−16^

To achieve optimal performance of immobilized proteins, various immobilization techniques have been developed based on covalent binding by reaction with the amino (−NH_2_) moiety^17,18^ or the use of a spacer to ensure better accessibility of the target molecules.^19^ In addition, oriented immobilization, which allows direct exposure of the protein’s active site, can be applied by exploring a specific protein structure, such as the thiol (−SH) antibody residues^20^ or, more commonly, by introducing reactive functional groups or tags on the protein via genetic engineering.^21−24^ The common goal of different immobilization strategies is to obtain immobilized protein with high biological activity, being dependent on its specific activity and amount. Therefore, to optimize the specific activity of the immobilized protein, determination of the amount of immobilized protein is of utmost importance.

The amount of immobilized protein can be determined by different techniques.^2,25−27^ Simple, indirect techniques, such as determination of solution depletion during immobilization by monitoring the protein concentration via UV absorbance at 280 nm, are the most common. However, this method can, in some cases, provide misleading values for the immobilized protein estimation, particularly if part of the immobilizing protein is nonspecifically adsorbing to the matrix and could be eluted when the mobile phase is changed, if the immobilizing protein agglomerates, or if it adsorbs nonspecifically to the confinement vessel.^28^ An alternative is the use of the cross-linker *N*-succinimidyl-3-(2-pyridyldithio)-propionate, which releases pyridine-2-thione upon reaction with cysteine (SH residue) and can be monitored spectrophotometrically.^29−31^ The advantage of this approach is that the stoichiometric amount of reacted groups can be monitored, allowing direct progress of immobilization but limited to proteins containing cysteine residues or requiring pretreatment.^31^ Moreover, the quantitative amount of immobilized protein molecules is sometimes difficult to estimate because they may be bound at multiple sites.^32^ Direct techniques, on the other hand, include destructive elemental analysis and protein hydrolysis in the acid at an elevated temperature^33^ or methods that contaminate the sample, such as dye binding^25^ and radiolabeling.^26^ In addition, there are several techniques for surface analysis, including scanning electron microscopy, transmission electron microscopy, X-ray photoelectron spectroscopy, surface plasmon resonance, circular dichroism spectroscopy, atomic force microscopy, and time-of-flight secondary ion mass spectroscopy.^34^ Besides complex and expensive equipment, a special type of matrix or a suitable sample pretreatment is required, which prohibits its daily use. The fact that determining the amount of immobilized protein is extremely important and yet challenging is demonstrated by a recently developed immobilization strategy using ultrafast immobilization chemistry in combination with genetical engineering.^35^ Due to the extremely high binding efficiency of recombinantly modified proteins with genetically introduced tetrazine moieties, resulting in almost complete immobilization of the available protein, it is assumed that the amount of immobilized protein is equal to the amount of protein in solution, making direct determination redundant. Clearly, a simple method to determine the amount of immobilized protein that can be applied to any type of support in a nondestructive and noninvasive manner would be beneficial for optimization of immobilization, resulting in cheaper production and better performance.

In this work, we propose a simple method for direct, nondestructive, and noninvasive detection of the immobilized protein amount. The method is based on a pH transition phenomenon that occurs when two mobile phases of the same pH but different ionic strengths are exchanged with a matrix bearing ion-exchange functionalities. It has been demonstrated that the duration and/or amplitude of the pH excursion that occurs during such a shift depends linearly on the amount of ion-exchange groups on the matrix.^36−40^ Recently, this approach was modified to allow the detection of immobilized protein A, a ligand commonly used for monoclonal antibody purification.^41^ Here, we demonstrate that this technique can be applied to various immobilized proteins and allows determination of their amount in flow-through mode without any pretreatment. Due to the use of biocompatible buffers, the performance of immobilized proteins is not affected. Therefore, the method can be used for the evaluation of immobilization efficiency and also of daily performance. Moreover, the sensitivity of the method can be adjusted by varying the buffer type, concentration, and pH. In addition, a mathematical model with solution algorithm is proposed, requiring only pre-existing data such as the amino acid sequence of the immobilizing protein, its molecular weight, and the immobilization matrix volume. The model was tested on the experimental data of various immobilized proteins. It was found that there is a linear range between the amplitude of the pH transition excursion and the amount of immobilized protein, assuring method constant accuracy.

## Materials and Methods

### Materials

Bovine serum albumin (BSA) (A2153), peroxidase from horseradish (HRP) (P8250), lysozyme (LYZ) from chicken egg white (62971), and glucose oxidase (GOX) from *Aspergillus niger* (G2133) were purchased from Sigma-Aldrich, protein A (protA) (10600—P07E) was from Sino Biological Inc., sodium carbonate (Na_2_CO_3_) was from Honeywell, sodium chloride (NaCl) and ammonium sulfate [(NH_4_)_2_SO_4_] were from Merck KGaA, hydrochloric acid (HCl) and sodium phosphate dibasic (Na_2_HPO_4_) were from Honeywell Fluka, and lactic acid (90% solution) was from Acros Organics.

High internal phase polymer (polyHIPE) matrix with a porosity of 80% bearing epoxide functional groups was prepared and characterized as described elsewhere.^42,43^

### Protein Immobilization and pH Measurements

Immobilization buffer for protA, BSA, and LYZ was 0.1 M Na_2_CO_3_/HCl, 0.5 M NaCl, pH 7.4. The proteins were dissolved in the buffer at concentrations ranging from 0.5 to 1.3 g/L to achieve different loadings of the immobilized protein. The polyHIPE matrix was placed in a housing and washed with the immobilization buffer until no change in UV absorbance (TECAN infinite M200Pro, Switzerland) was detected at the housing outlet. Afterward, the housing was disassembled and dried with compressed air, while the excess solution from the polyHIPE matrix was wiped. After reinserting the polyHIPE matrix into the housing, 1 mL of the protein solution was pumped through the matrix for 3 h at RT using a syringe on each side. The pore volume inside the polyHIPE matrix was considered for calculation of the initial protein concentration in solution. The amount of immobilized protein was calculated from the difference in the protein concentration before and after immobilization, as determined by UV absorbance at 230 nm and the corresponding calibration curve. Immobilization buffer for GOX and HRP was 10 mM Na_2_HPO_4_/HCl, 1.5 M (NH_4_)_2_SO_4_, pH 7. The immobilization procedure was the same as described above, except that the incubation period was 5 days.

The pH transition method described in detail elsewhere^41^ was performed for all immobilized systems with lactate buffer at pH 4.3 (for protA, BSA, and LYZ) or at 3.9 (for GOX and HRP) and 1 mM concentration, unless otherwise indicated. To check the reproducibility, each measurement was performed in three replicates, and the RSD value of the peak height was calculated. The results for protein A (Figure S5) show a close match of all three profiles with the height RSD below 5%.

### Theoretical Background and Mathematical Modeling

The model used to predict pH gradients for protein-immobilized matrix is an extension of the models developed by Vetter et al.^44^ and Pabst et al.^38,39,45^ for matrix-bearing ion-exchange groups. The developed framework was adopted for immobilized proteins, which are also weak ion-exchangers due to their amino acid composition. Several proteins were examined (Table S1). The mathematical model considers both the solution and ion-exchange equilibria along with the mass balances and solves the conservation equations. The main assumptions of the proposed model, whose mathematical framework and theoretical background are described in detail in the Supporting Information, are as follows: (i) The concentration of the immobilized protein is constant and uniformly distributed throughout the matrix; (ii) only charged amino acid residues (acidic: aspartic acid, glutamic acid, cysteine, tyrosine, C-terminal; basic: lysine, arginine, histidine, N-terminal) participate in the ion-exchange process according to the pH of the solution and the individual p*K*_a_ values of the residues; (iii) for simplicity, nonretention of buffer species is considered; (iv) the mass transfer of counterions and buffer species is rapid, so that a local equilibrium between the mobile phase and the stationary phase is established for these species; (v) immobilization does not affect the amount of ionizable protein residues.

Mathematical model is described in the Supporting Information with key equations presented in the Results and Discussion section (verification of the mathematical model). For a detailed theoretical background of the equilibrium equations, the reader should refer to the original papers.^46−48^

## Results and Discussion

Due to the importance of protein immobilization, a variety of different methods have been developed to determine the amount of immobilized protein.^2,25−27,29−34^ Nevertheless, there is a lack of a direct method that can be implemented in a noninvasive manner, allowing inspection of a particular sample without affecting its properties. As recently demonstrated, the pH transition method, which allows determination of the amount of ionizable groups on the matrix,^37,38,40,41,49^ can also be used for the detection of immobilized protein A. Therefore, we investigated whether a modified method could be applied to a specific immobilized protein and whether its amount could be determined accurately. The method is based on the pH change when the matrix of interest is subjected to a stepwise change in buffer ionic strength. In a flow-through mode, the pH transition represents an intermediate state resulting from the difference in velocity between retained and nonretained ions. This is due to an established ion-exchange equilibrium between ions in solution and ions on the matrix surface, which depends on the dissociation equilibria of the ionizable groups.^38,39,44,45^ The magnitude and shape of the pH shift is influenced by the mobile phase composition and pH value, as well as by the type and quantity of ionizable groups immobilized on the matrix. Therefore, the pH transition profile can be directly correlated with the amount and type of ionizable groups.^38^ Since all ionizable groups present on the matrix contribute to the pH transition, this method is not limited to the flat surfaces like many direct techniques^25−27^ but can be readily applied to all proteins immobilized on porous materials, providing information about the entire immobilized protein. Since the method requires only biocompatible buffers that can be adjusted to the pH stability range of the protein, its application does not affect the protein biological activity. To test the generality and sensitivity of the method, several proteins were immobilized and studied. Two of them are frequently used enzymes for the detection of glucose and hydrogen peroxide, namely, GOX and HRP, which are also commonly used as biodetectors and biosensors,^50,51^ protA, a frequently used affinity ligand,^41^ BSA, a protein applied usually to reduce nonspecific interactions,^52^ and LYZ, an enzyme with a very high isoelectric point.^53^ All selected proteins were immobilized in different amounts on the porous polyHIPE matrix (see Materials and Methods section for details).

Initially, we determined the amount of immobilized protein by measuring the UV absorbance of the solution before and after immobilization. Independently, we verified the absence of nonspecific adsorption on the housing (the immobilization procedure without the polyHIPE matrix was performed), so it can be assumed that the change in the solution protein concentration was due to its immobilization or adsorption on the matrix. In addition, after immobilization was completed and the solution containing residual non-immobilized protein removed, the polyHIPE matrix was exposed to different solutions (distilled water and buffer solution containing 1.5 M NaCl) to potentially desorb the nonspecifically adsorbed protein. No change in solution absorbance after washing indicated that the entire protein was irreversibly immobilized to the matrix, and the change in solution concentration during immobilization can be considered as an estimate of the amount of immobilized protein. Once washing was completed, pH transition experiments were performed. Although we are free to choose the buffer for a particular immobilized protein based on its buffering capacity,^41^ for simplicity, all experiments were performed with a single type of buffer (lactate) at two different pH values (3.9 and 4.3).

### Verification of the Mathematical Model

The pH transition was performed experimentally and predicted based on the proposed mathematical model (for full derivation, see the Supporting Information). The latter was constructed as a flow-through model considering mass balance, convection, and dispersion in axial direction and species adsorption
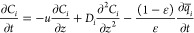
1

For the nonretained components, such as buffers,  and local equilibrium are assumed, while for retained components, such as sodium and chloride ions, , and *q*_*i*_ can be calculated based on
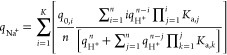
2
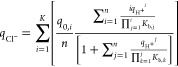
3

**Figure 1:**
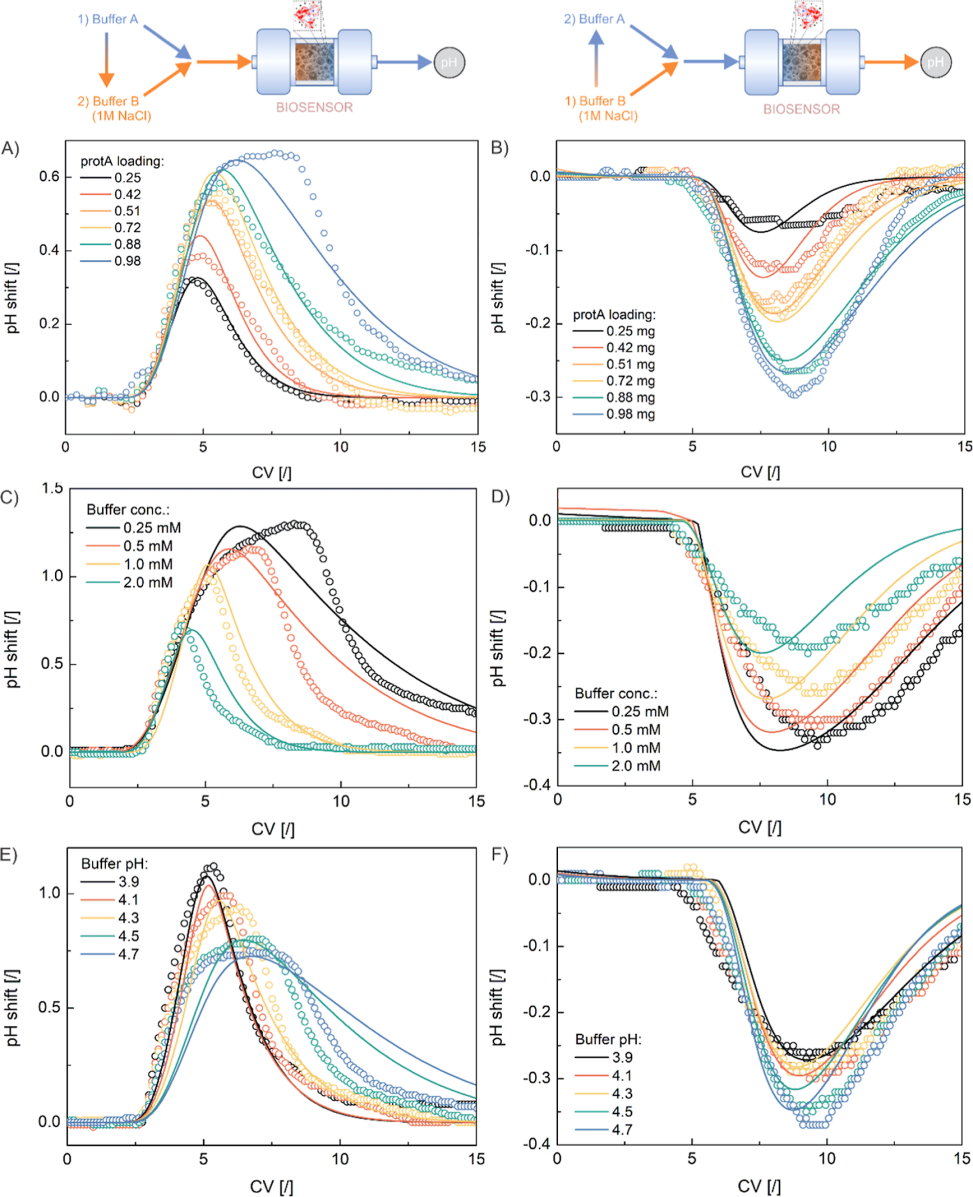
Comparison of experimental (circles) and model-based (line) pH transition profiles for polyHIPE samples containing various amounts (0.25, 0.42, 0.51, 0.72, 0.88, and 0.98 mg) of protA (A-B), and samples containing 0.98 mg of immobilized protA using different lactate buffer concentrations (0.25, 0.50, 1, and 2 mM) at pH 3.9 (C,D) and using different pH values (3.9, 4.1, 4.3, 4.5, and 4.7) of 1 mM lactate buffer (E,F). The pH responses for stepwise change from buffer A to buffer B (A,C,E) and from buffer B to buffer A (B,D,F) are presented together with a schematic demonstration of the experiment. Buffer A: 1 mM lactate buffer, pH 4.3; buffer B: 1 mM lactate buffer containing 1 M NaCl, pH 4.3; flow rate 3 mL/min.

Considering the solution and matrix electroneutrality along with the Donnan equilibrium, the mass balance and resulting pH profiles can be calculated numerically. Initially, the pH transitions for the stepwise buffer change on a matrix containing different amounts of immobilized protein A were used to test the mathematical model (Figure 1A,B). Note that the pH shift of the untreated matrix (matrix exposed to the immobilization procedure but without the dissolved protein) was subtracted from the individual experimental data to estimate only the pH changes caused by the immobilized protein. All pH profiles showed a positive shift for the stepwise change from buffer A to buffer B and a negative shift for the stepwise change in the other direction. According to the isoelectric point of protein A (∼5.1), its net charge, at pH 4.3, is positive. Therefore, one would expect pH transition profiles similar to those of anion exchange groups, for which a pH increase with the increase in the buffer ionic strength and vice versa has been reported.^40^ In fact, such a trend was correctly predicted by a mathematical model. Furthermore, the higher the amount of immobilized protein A, the more pronounced the pH shift. Both the width and, to some extent, the height of the peaks increased with increasing mass of immobilized protein. The model correctly predicted the magnitude and, to a greater extent, the shape of the pH transition for both stepwise changes. It should be emphasized that all model calculations were based only on the pre-existing data (buffer formulation, amount of immobilized protein, protein amino acid sequence, matrix porosity, and dimensions) and mechanistic description (dissociation mechanism, column equilibrium, and electroneutrality assumption) without any model parameters fitted to experimentally measured pH profiles. The amino acid sequence and the mass of the immobilized protein provide the amount of charged ionizable groups, which, considering the theoretical p*K*_a_ value for residues, defines the extent of ionic character of the immobilized protein. On the other hand, the process dynamics is accounted through equilibrium assumptions for the buffer and the matrix as well as by the plug flow regime. Good agreement between experimental data and simulated profiles confirms the validity of the chosen assumptions and calculation procedure to correctly predict the pH shifts for immobilized protein A.

Since promising results were obtained with protein A, the general applicability of both the pH transition method and the proposed mathematical model was further tested on other immobilized proteins. For this purpose, BSA, LYZ, GOX, and HRP were used. Despite the differences in the protein structure and even more in their biological function, similar pH profiles to protein A were obtained, namely, positive shifts when switching stepwise from buffer A to buffer B and negative shifts when switching backward (Figure S6). As mentioned before, the pH value of all immobilized proteins was below their isoelectric point, proving that the overall net charge of the protein was positive, and therefore the pH transition mimicked the response of the anion-exchange resin. In addition, all pH profiles showed a so-called chromatographic peak type,^40,41^ the shape and magnitude of which strongly depend on the amount of immobilized protein and the buffer used. Still, the prediction based on the mathematical model matches closely with the experimental data. Of course, there is some data scattering, but the experimental errors are probably due to a very small pH transition shift (below 0.1 unit). In general, however, the good match between the experimental and predicted results indicates the generality of the proposed theoretical approach to correctly predict the pH response as a function of immobilized mass for various proteins.

### Effect of Buffer Concentration and pH Value

Since buffer concentration and pH have a significant effect on the duration and magnitude of the pH transition,^37^ their influence was investigated. Initially, different buffer concentrations (0.25, 0.5, 1, and 2 mM) were tested (Figure 1C,D). It has been reported^36,37,41^ that high buffer concentrations have little effect on the pH shift profile, while low buffer concentrations can drastically alter the pH deviation. A similar trend was observed here. The lower the buffer concentration, the higher and longer the pH shifts, which can be attributed to a decrease in the buffer capacity. The buffer concentration can therefore be used to obtain an optimal pH shift profile for the particular sample: an excessively long duration of the pH transition is rather impractical for routine measurements, while on the other hand, the pH shift should still be sufficient to accurately detect the amount of immobilized protein. Therefore, a reasonable compromise between duration and magnitude of the pH response must be chosen.

Importantly, the model simulation accurately predicts both the magnitude and shape of the pH transition for a stepwise change from B to A. However, the experimental profiles and the model prediction for the stepwise change from A to B begin to diverge when the buffer concentration is below 1 mM. We assume that as the buffer concentration decreases, the influence of an additional mass-transfer resistance, such as the electric double layer, becomes more pronounced. At lower ion (buffer) concentration, the electrostatic interactions between charged groups on the protein surface and in the bulk solution intensify and the diffuse layer becomes thicker.^54^ Accordingly, additional resistance to ion exchange occurs, which affects the shape of the pH profile. As this mechanism was not considered in the mathematical model, discrepancy between the experimental and simulated profiles arises. However, since the buffer concentration and pH values can be easily adjusted to obtain the chromatographic peak shape as well as to avoid the introduction of additional model parameters that are sometimes difficult to measure, the simple mathematical model was preserved.

In the next set of experiments, the concentration of the buffer was kept constant (1 mM), while its pH value varied from 3.9 to 4.7 (Figure 1E,F). During the stepwise change from A to B, a lower pH value enhanced but shortened the pH shifts. As pH increases, the overall net charge of protA changes, leading to a change in its ionic character. As a result, flat-top peaks are detected, with a quasi-steady state (an equilibrium) likely to occur at a pH plateau. pH of the implemented buffer can therefore be used to affect the shape of the pH profile to become narrower or wider. On the other hand, relatively similar pH profiles for the stepwise change in the other direction were obtained for all formulations. We assume that this is because in the pH range between 3.6 and 4.5 (which is relevant for the change from buffer B to A, also considering the observed pH shifts) both protein and buffer capacities decrease relatively proportionally (in relation to each other) with the changing pH value. Therefore, changes in buffer pH do not drastically affect the mechanism, resulting in similar pH profiles. Nevertheless, for all selected conditions, the model correctly predicted the magnitude and, apart from stepwise change from buffer A to buffer B with pH 4.5 and 4.7, also the profile shape. A similar discrepancy between the experimental data and the model prediction for very low buffer concentrations (Figure 1C) is observed, again likely due to the simplicity of the proposed model. Accordingly, to accurately predict the shape of the pH profile with the proposed model, conditions that provide chromatographic peaks are preferred. Additional reason to evaluate the chromatographic peak shape is method sensitivity, which was demonstrated to be higher as for flat-top peaks.^40^

### Evaluation of pH Profiles for Estimation of Immobilized Protein Amount

**Figure 2:**
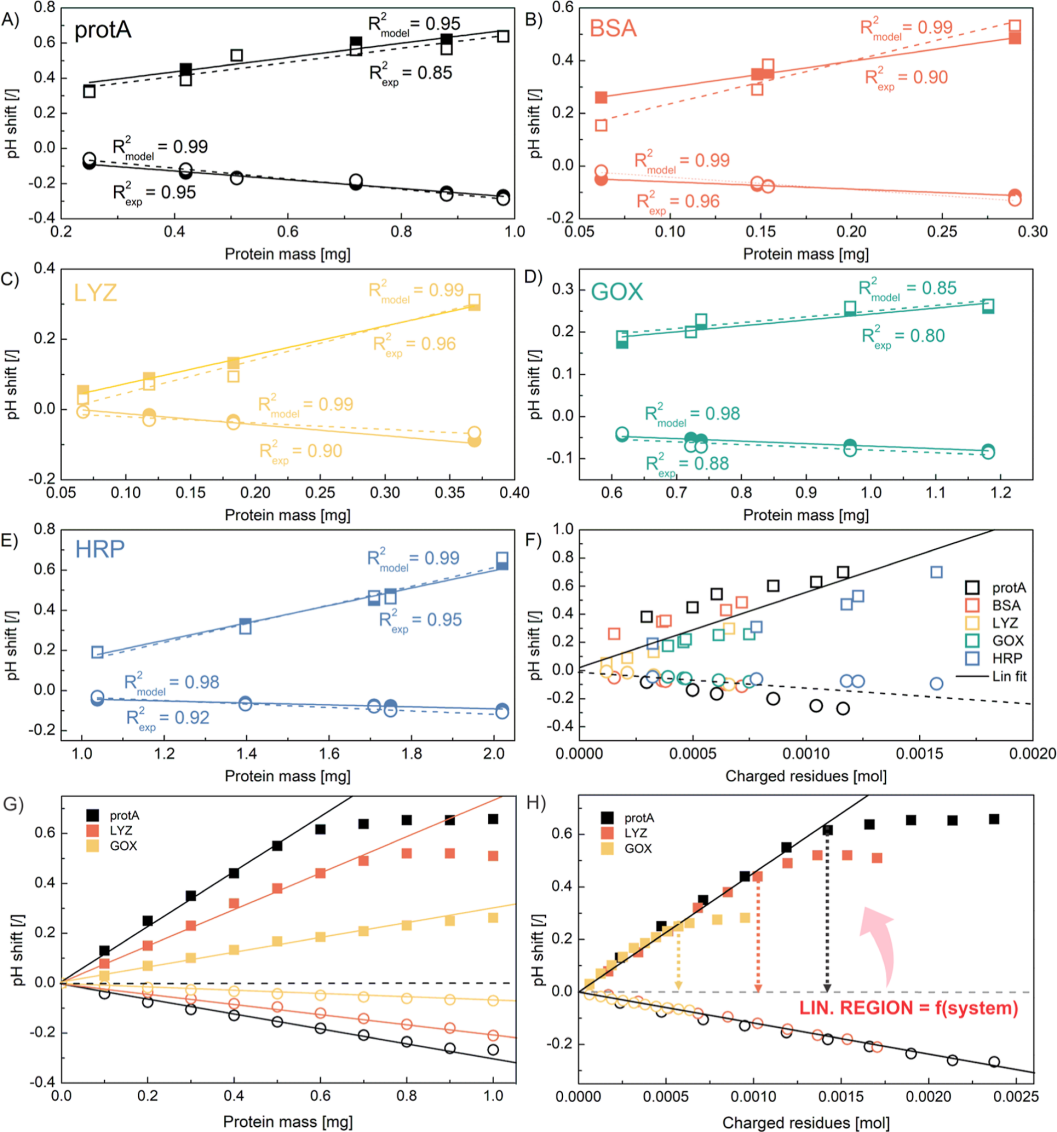
Correlation between the pH transition peak height and the amount of immobilized protein for both stepwise changes for protA (A), BSA (B), LYZ (C), GOX (D), and HRP (E). Note that the solid lines and symbols represent the model-based data (correlated with the *R*_model_^2^ value), whereas the dashed lines and open symbols represent the experimental data (correlated with the *R*_exp_^2^ value). The magnitude of the experimental pH shift plotted against the moles of charged residues (F). The model-based height of the pH transition peak as a function of the amount of immobilized protein (G) and the model-based overall charged residues (H) for protA, LYZ, and GOX.

As discussed in our previous work,^40^ the magnitude of the pH peak can be correlated with the amount of immobilized protein by determining either the full width at half-maximum (fwhm) or the height (ΔpH) of the peak for the flat-top or chromatographic peak shape, respectively. Since chromatographic peaks were predominant in our experiments, a linear correlation between the height of the pH transition peak (ΔpH) and the amount of immobilized protein was investigated. Accordingly, the values of the pH peak height were plotted against the amount of immobilized protein. A good linear correlation was found for both experimental and model-based data (Figure 2A–E), with the experimental data showing a higher scatter. The same analysis was performed correlating fwhm with the mass of immobilized protein, but, expectedly, poorer linearity was obtained (data not shown).

So far, the proposed method has demonstrated that there is a linear correlation between the pH response and the mass of the different immobilized proteins. However, a different sensitivity was found for each protein. Of course, each protein has a unique amino acid sequence with a different number and type of charged residues (Table S1). In addition, the molecular mass of the protein also varies. Therefore, it seems more reasonable to compare the pH shift with the total number of immobilized charged groups. This can be estimated by dividing the immobilized protein mass by its molecular mass and multiplying it with the number of charged residues of the protein molecule at a given pH. For this purpose, the amount and degree of dissociation of the relevant amino acid residues must be considered along with their p*K*_a_ values and local pH (note that during the calculation procedure of the mathematical model, all these calculations are constantly iterated until they converge). Calculations were performed for each protein and plotted against the maximum peak height (ΔpH) based on the experimental data. The results presented in Figure 2F show a fairly good linear trend.

### Theoretical Evaluation of Immobilization Impact and Method Linearity Range

Since the mathematical model correctly predicted the direction, magnitude, and shape of pH profiles, the latter especially for chromatographic peak shape profiles, its usability as a process monitoring tool was investigated. Importantly, the magnitude of individual pH shifts could be predicted based solely on the buffer properties, the mass of the immobilized protein, and its amino acid sequence in free (native) form. This highlights the generality of the proposed method as the type of pH response for a particular protein can be assessed based on its sequence alone. Furthermore, since the sequence of the protein in a free form was used, the effect of immobilization seems to be negligible. This was confirmed by investigating the effect of covalent binding and denaturation, demonstrating almost no changes in the pH profile (Supporting Information—effect of covalent binding and denaturation on the pH profile).

As shown in the previous section, most experimental and simulated results exhibited a chromatographic peak shape for which a linear correlation between the height of pH shift and the mass of the immobilized protein (or more generally, the amount of immobilized charged groups) was found. Therefore, we investigated if such a correlation is always present or there is an amount above which the nonlinearity occurs. Model simulations were used to investigate the range of linearity for protA, LYZ, and GOX (Figure S7) as they differ significantly in molecular weight and isoelectric point. The simulated immobilized mass of each protein ranged from 0.1 to 1.0 mg. The summarized results shown in Figure 2G indicate that the magnitude of pH shifts increases linearly with the immobilized mass over the entire range tested for negative pH shifts (having smaller magnitude), whereas for positive pH shifts, it followed a linear trend until a certain plateau is reached. Thereafter, the magnitude of the pH shift remained fairly constant, while the duration of the pH shift continued to increase (see also simulation in Figure S7), confirming the experimental results for ion-exchange groups.^38−40,44,45^ Different slopes were observed for each protein (Figure 2G) indicating a different sensitivity of the method to the mass of the immobilized protein. However, when the number of moles of charged residues was plotted instead of the protein mass, a nearly perfect overlap was obtained for the linear part (Figure 2H), with the linearity range of the individual protein also clearly identified. Under the conditions studied, linearity is observed up to 0.0006 mol for GOX (yellow arrow), whereas it extends to 0.0015 mol for protA (black arrow).

Since a linear response is preferred to ensure constant accuracy of the method, the effect of buffer composition (concentration and pH) on a linear range was investigated next. Interestingly, buffer concentration does not affect the linear range (Figure S8A), but it does affect the sensitivity of the method (the slope of the line changes). On the other hand, changing the buffer pH affects both the sensitivity and the linear range (Figure S8B). The closer we get to the isoelectric point of the protein (at higher pH), the lower the ionic character, resulting in a lower pH response. A similar trend was observed in Figure 1E,F.

**Figure 3:**
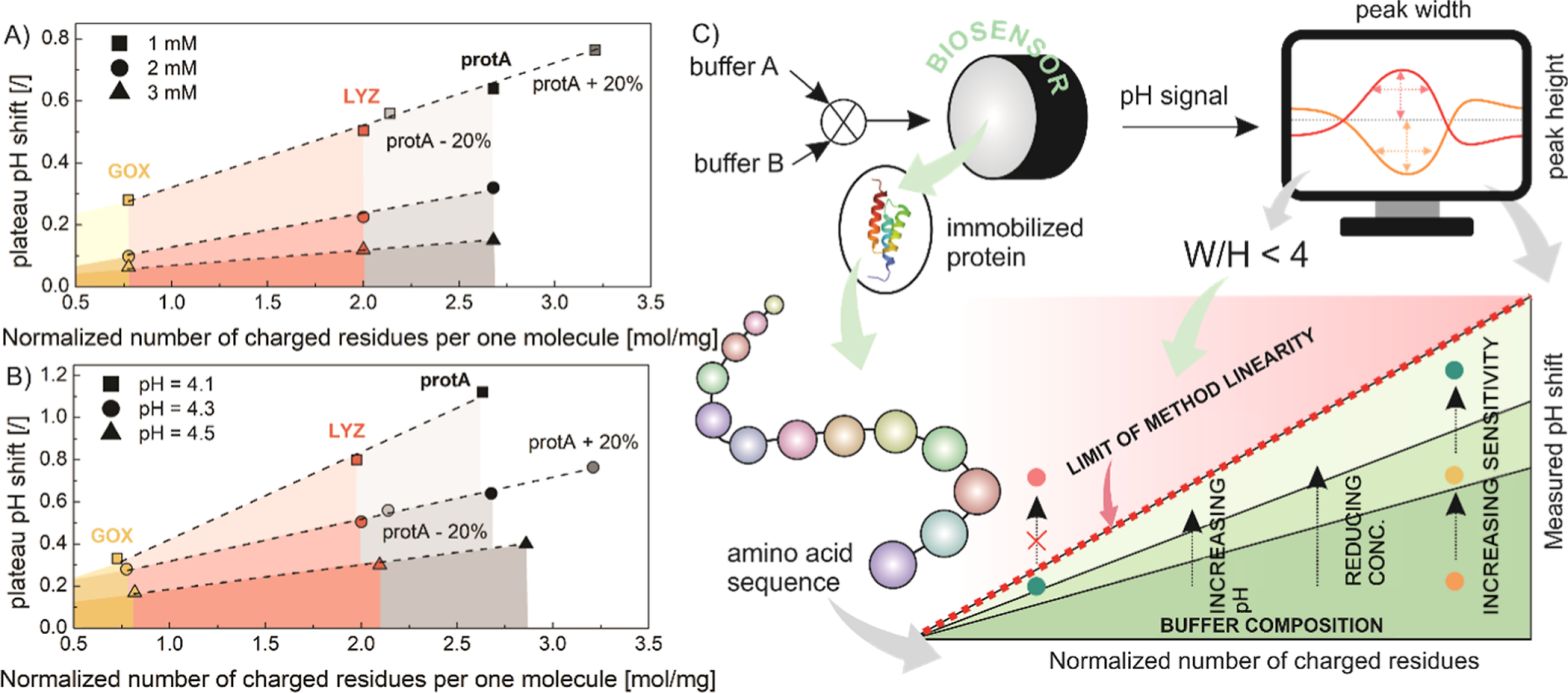
Relationship between the plateau pH shift and the total number of charged residues in a protein molecule, normalized to protein molecular weight as a function of buffer concentration (A) and buffer pH (B). Note that protA + 20% and protA – 20% represent the simulation for the protA system, with the molecular weight increased and decreased by 20%, respectively. The colored region represents the region where a linear correlation is expected, meaning the method/model can be applied with constant accuracy. The generalized solution algorithm for assessing the amount of protein immobilized (C).

Despite the observed trend, it is still not clear how to estimate the linearity range, although it is of utmost importance to accurately determine the amount of immobilized protein based on the pH response. Naturally, this can be evaluated by running model simulation for a particular protein or even by constructing a calibration curve from experimental data by immobilizing different amounts of the target protein and measuring its pH response. Nevertheless, it would be more convenient to estimate, especially during optimization of the pH transition method for a particular application, if a single measurement is within the linear range. So far, the analysis has considered only the total number of immobilized charged residues without taking into account their distribution. Unlike a functionalized matrix in which the charged groups are uniformly distributed, for immobilized proteins, they are localized on the protein molecule, and therefore their local density may depend on the protein charge and mass. For this reason, we investigated whether the upper limit of the linear range could be correlated with the number of charged residues (at a given pH) on a single protein molecule, normalized to the molecular weight of the protein. When the simulation data of the pH shift plateau onset (upper limit of the linear range) were plotted against the normalized charged residues, a perfect linear relationship was obtained (Figure 3A,B). To further confirm this correlation, protA simulations were performed assuming 20% higher and lower molecular mass (keeping the number of charged groups constant). The results indicate that the plateau onset and thus the upper limit of the linear range can be estimated from the charge density. As expected, the obtained linear slope changes when the buffer concentration and pH are different. Although each protein has a different maximum pH shift, a similar response to changes in buffer composition was observed. Considering all this, only buffer formulations (pH, concentration) that lie below (or at) the linear threshold (highlighted in color in Figure 3A,B) provide a linear dependence between the pH response and the immobilized mass of a particular protein.

Once such correlation is constructed, one can predict the linear range of a newly immobilized protein under the same buffer conditions. An alternative approach, which would require a single measurement, is to evaluate the linear range from a peak shape, more specifically, (a) peak symmetry. Due to a discrepancy between the simulated and experimentally obtained profiles with a flat-top shape, the symmetry of both simulated and experimentally measured pH peaks was investigated. For simulated pH peaks, it was found that there is a linear dependence between the height of the pH peak and the immobilized mass for peak asymmetry below 2.0 (Table S2). Note that the peak asymmetry was determined at half the height of the individual pH peak. On the other hand, analysis of the experimental data (performed for all proteins tested) revealed that the linear range is preserved when the ratio between the pH peak width at 90% of the peak height and the pH peak height is less than 4 (Table S3). If, under certain buffer conditions, the measurement falls outside the linear range, the buffer pH should be changed and the pH transition method repeated until the proposed criterion is met as schematically presented in Figure 3C and explained in detail in the Supporting Information (solution algorithm and limit of detection).

High biological activity obtained with a low amount of immobilized protein is a common optimization criterion during the development of an immobilization protocol. While methods for determining the specific biological activity of proteins in solution are well established already prior to their immobilization, determining the amount of immobilized protein is often a bottleneck for determining the specific biological activity of the immobilized protein. As the proposed method eliminates this challenge, it facilitates the optimization of immobilization protocols but also allows to discriminate if, for example, change in biological activity during prolonged use is a consequence of protein leaching or ligand contamination.^55,56^ Being biologically compatible, it can therefore be implemented for in-process monitoring.

## Conclusions

The developed method allows simple, direct, noninvasive determination of the immobilized protein amount without affecting its biological properties. As such, it can be implemented during preparation and optimization of novel devices based on immobilized proteins such as, for example, biosensors or affinity columns, for their quality control and also as an in-process monitoring tool. Due to the implementation of biologically compatible buffers and standard laboratory equipment consisting solely of a pump and a pH sensor, it can be easily validated and therefore used under good laboratory and manufacturing practices. For this reason, it is an attractive option for the laboratory as well as industrial environments, where devices involving immobilized proteins are implemented.

## Supporting Information Available

Developed framework for the proposed mathematical model, effect of immobilization, solution algorithm, limit of detection along with additional pH transition profiles (experimental and model-based), and extracted details (PDF)
