# Supplementary material for: Rapid, Direct, Noninvasive Method to Determine the Amount of Immobilized Protein
Source: Anal Chem. 2023 Mar 20;95(13):5643–51. doi: 10.1021/acs.analchem.2c05402 (PMC10077329; doi:10.1021/acs.analchem.2c05402)
Supplement: Supplementary file 1 — ac2c05402_si_001.pdf [file ac2c05402_si_001.pdf]

Supporting Information

Rapid, direct, non-invasive method to determine the amount of immobilized protein

Rok Ambrožič†, Rok Mravljak†, Aleš Podgornik†,††*

† University of Ljubljana, Faculty of Chemistry and Chemical Technology, Večna pot 113, 1000 Ljubljana, Slovenia

†† COBIK, Mirce 21, 5270 Ajdovščina, Slovenia

* correspondence: Aleš Podgornik, Faculty for Chemistry and Chemical Technology, University of Ljubljana, Večna pot 113, 1000 Ljubljana, Slovenia

Tel.: +386 1 479 8584

E-mail: ales.podgornik@fkkt.uni-lj.si

The file incudes mathematical framework and theoretical background of the proposed mathematical model, additional experimental and model-based simulations of pH profiles, and additional information collected in tabular form extracted from the raw data.

Table of content

- The developed framework for proposed mathematical model. S-2
- Calculations and solution routine S-5
- Effect of covalent biding and denaturation on pH profile S-7
- Solution algorithm and limit of detection S-9
- Figure S1: Schematic diagram of the pH transition measurement. S-5
- Figure S2: Effect of number of lysine residue on the pH transition profiles for samples containing immobilized LYZ and protA. S-7
- Figure S3: Effect of conformational changes of the protein pH profile. S-8
- Figure S4: The limit of detection of proposed method. S-11
- Figure S5: Robustness of the method. S-12
- Figure S6: Experimental and model-based pH transition profiles for samples containing immobilized BSA, LYZ, GOX and HRP. S-13
- Figure S7: Model-based pH transition profiles as a function of immobilized masses for protA, LYZ and GOX. S-14
- Figure S8: Effect of buffer composition on the linear region of protA. S-15
- Table S1: Summary of immobilized protein properties. S-16
- Table S2: The pH peak asymmetry as a function of the immobilized mass of the protein. S-16
- Table S3: The peak height and peak width ratio of tested pH peak. S-17
- References S-18

1. The developed framework for proposed mathematical model:

To account for solution equilibria, the charged species must satisfy the electroneutrality condition at a given pH:

$$C_{H^+} + C_{Na^+} + \sum_{i=1}^{M} iC_b^{i+} = \frac{K_W}{C_{H^+}} + C_{Cl^-} + \sum_{j=1}^{N} jC_a^{j-} \tag{1}$$

where K_w is the ionic product of water, C_b^{i+} and C_a^{j-} are the concentrations of the charged basic and acidic buffer species, respectively, and M and N are the valence of the charged basic and acidic buffer species, respectively. The concentration of the charged buffer species can be calculated by considering the dissociation equilibrium. For an acidic buffer (as in our study) with valence N_i, the concentration of each buffer species is given by:

$$C_a^{1-m} = \frac{C_{TOT} \prod_{i=1}^{m-1} \frac{K_{a,i}}{C_{H^+}}}{1 + \sum_{i=1}^{N_i} \left(\prod_{i=1}^{m-1} \frac{K_{a,i}}{C_{H^+}}\right)} \tag{2}$$

for $m = 1,2\ldots N_i$

where C_{TOT} is the total buffer concentration, N_i is the ion valence, and $K_{a,i}$ is the deprotonation constant for individual buffer species. Eq 2 can be adopted also for basic buffers with only minor modifications[1, 2]. Note that the activity coefficients are required to calculate the correct dissociation constants and can be determined according to the Davies equation[2]. Subsequently, eq 1-2 can be solved numerically for C_H^+ which allows the calculation of the pH.

Two types of amino acid residues may be present on immobilized protein molecules, namely weak acid groups and weak base groups. The acid groups dissociation is described as:

$$RH \rightleftharpoons R^- + H^+ \tag{3}$$

where

$$K_{a,i} = \frac{q_{R^-} q_{H^+}}{q_{RH}} \tag{4}$$

is an apparent dissociation constant for acidic groups and can be estimated from the residual pKa(s) (Table S1). Likewise, basic groups dissociation is described as:

$$R + H^+ \rightleftharpoons RH^+ \tag{5}$$

where

$$K_{b,i} = \frac{q_R q_{H^+}}{q_{RH^+}} \tag{6}$$

is an apparent dissociation constant for basic groups and can be estimated from the residual pKb(s) (Table S1). The q_i are the concentrations of protonated and deprotonated functional groups present in the respective (acidic or basic) matrix. To account for the matrix electroneutrality, the following condition must be satisfied:

$$q_{Cl^-} + q_{OH^-} + q_{R^-} = q_{Na^+} + q_{H^+} + q_{RH^+} \tag{7}$$

In practice, it turns out that both q_{OH^-} and q_{H^+} are usually negligible[1, 3, 4], while only one of q_{R^-} and q_{RH^+} is relevant at certain pH values (depending on the charge distribution of the proteins). By combining eq 3-7, we obtain a general approximation for sodium ions:

$$q_{Na^+} = \sum_{i=1}^{K} \left[\frac{q_{0,i}}{n} \frac{\sum_{i=1}^{n} i q_{H^+}^{n-i} \prod_{j=1}^{i} K_{a,j}}{\left[q_{H^+}^{n} + \sum_{j=1}^{n} q_{H^+}^{n-j} \prod_{k=1}^{j} K_{a,k} \right]} \right] \tag{8}$$

and/or chloride ions:

$$q_{Cl^-} = \sum_{i=1}^{K} \left[\frac{q_{0,i}}{n} \frac{\sum_{i=1}^{n} \frac{i q_{H^+}^{i}}{\prod_{j=1}^{i} K_{b,j}}}{\left[1 + \sum_{j=1}^{n} \frac{q_{H^+}^{j}}{\prod_{k=1}^{j} K_{b,k}} \right]} \right] \tag{9}$$

when NaCl is used to change buffer ionic strength, where q_0 is the total concentration of the individual weak acid or base functional groups (residues) on the protein molecule, K_a, and K_b are the dissociation constants for acid and base residues, n is the number of groups capable of parallel dissociations (in our case typical n = 1), and K is the number of different ionic groups/residues that can be protonated. In the case studied, this is also the number of different charged residues. Finally, solution and phase equilibrium can be related by the Donnan equilibrium:

$$q_{Na^+} C_{H^+} = q_{H^+} C_{Na^+} \tag{10}$$

$$q_{Cl^-} C_{OH^-} = q_{OH^-} C_{Cl^-} \tag{11}$$

where we assume that anions and cations are exchanged unrelatedly, yet highly dependent on solution and matrix pH value. For a given initial values of C_{H}^{+}, C_{Cl}^{-}, C_{Na}^{+} and C_{TOT} the q_{Na}^{+} and/or q_{Cl}^{-} can be calculated using eq 1-11. Note that q_0 is directly related to the concentration of the immobilized protein, while K_a and n are determined by the nature of the immobilized protein via its amino acid sequence.

2. Calculations and solution routine

Since the pH transition is a dynamic phenomenon, it was studied in a flow-through mode (Figure S1).

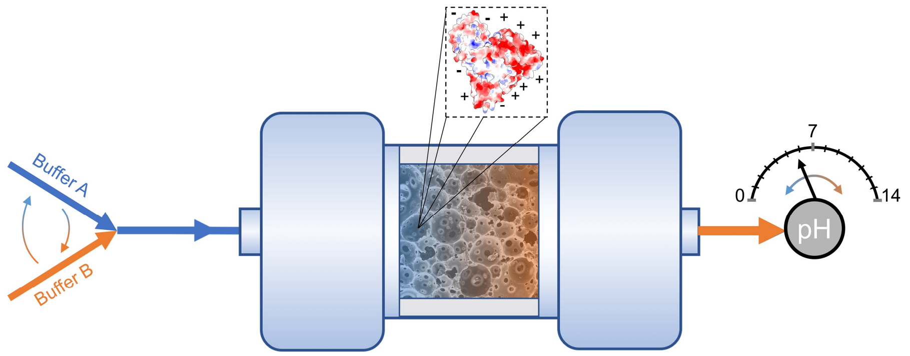

Figure S1. Schematic diagram of the pH transition measurement. Two buffers of same pH but different ionic strengths are exchanged at the inlet and flow through the porous polyHIPE matrix bearing immobilized protein. The pH is constantly recorded at the outlet.

Furthermore, a porous matrix was considered for protein immobilization, as being frequently used to increase the specific surface area, resulting in a higher amount of immobilized protein and thus a higher conversion or binding capacity. Due to an open-pore structure and flow regime, the material balance model describing the plug flow was used to predict the dynamics of the individual component:

$$\frac{\partial C_i}{\partial t} = -u\frac{\partial C_i}{\partial z} + D_i\frac{\partial^2 C_i}{\partial z^2} - \frac{(1-\varepsilon)}{\varepsilon}\frac{\partial \bar{q}_i}{\partial t} \tag{12}$$

where z is the axial coordinate of the matrix, u is the superficial velocity, ε is the open matrix porosity, and D_i is the axial dispersion coefficient. For the non-retained components, such as buffers, $\bar{q}_i = \varepsilon_p C_i$ and local equilibrium is assumed, while for retained components, such as sodium and chloride ions, $\bar{q}_i = \varepsilon_p C_i + (1-\varepsilon_p)q_i$, and q_i can be calculated based on eq 8-11.

A numerical solution of the conservation equations (12) was obtained by discretizing the axial derivative using forward finite differences and solving the resulting set of ordinary differential equations in time with built-in solvers in the MATLAB library. Numerical dispersion caused by the discretization was minimized by increasing the number of discretization points. All calculations were performed in MATLAB R2016b on a regular PC, with all results obtained within minutes.

3. Effect of covalent biding and denaturation on pH profile

Initially the simulations in which one of the lysine residues was omitted, assuming that it reacted with the matrix during immobilization (which is a commonly applied mechanism), was performed to check the effect of covalent biding on pH profile. No obvious difference was observed in the protA simulation, whereas only a slight deviation was observed in the LYZ simulation (Figure S2). This can be attributed to the fact that LYZ has a smaller number of charged residues per molecule, implying that the relative contribution of a single amino acid residue is higher. Nevertheless, the insignificant difference suggests that the original protein amino acid sequence available in the database can be used to predict the pH shifts of the immobilized protein. In addition, protein denaturation tests were performed to evaluate possible effects of conformational changes on pH profiles. A standard procedure with urea solution was performed[5] (samples without and with 0.979 mg immobilized protein A were transferred into 8 M urea for 1h at 60°C) and the pH profiles were evaluated before and after exposure. Since no obvious difference was observed (Fig. S3), we can conclude that protein denaturation does not affect the pH transition profile.

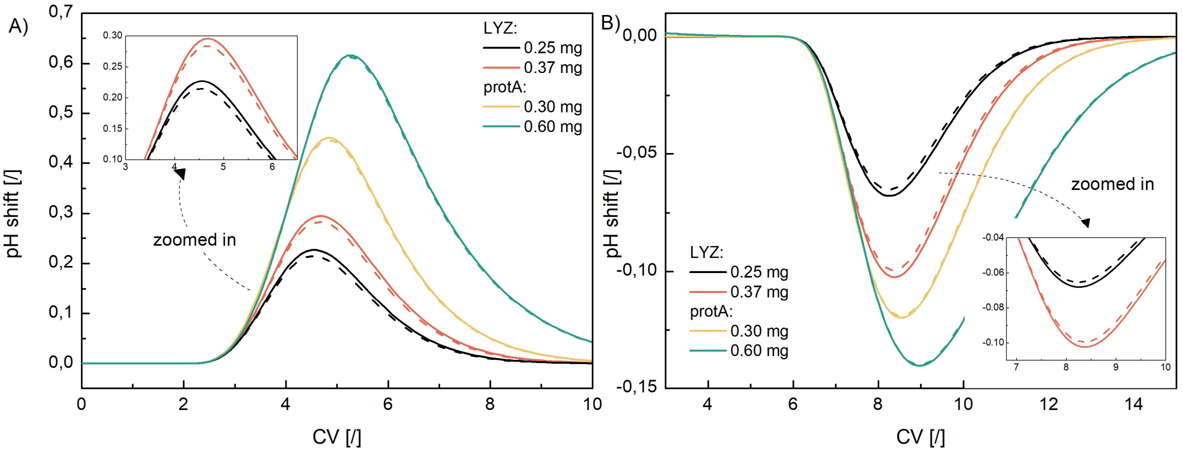

Figure S2. Model-based pH transition profiles for samples containing immobilized LYZ (0.25 mg and 0.37 mg) and protA (0.30 mg and 0.60 mg) for stepwise change from buffer A to buffer B (A) and buffer B to buffer A (B). The dashed lines represent the same starting material as the

solid line reduced by one lysine unit, as if it had been used for immobilization on the stationary phase.

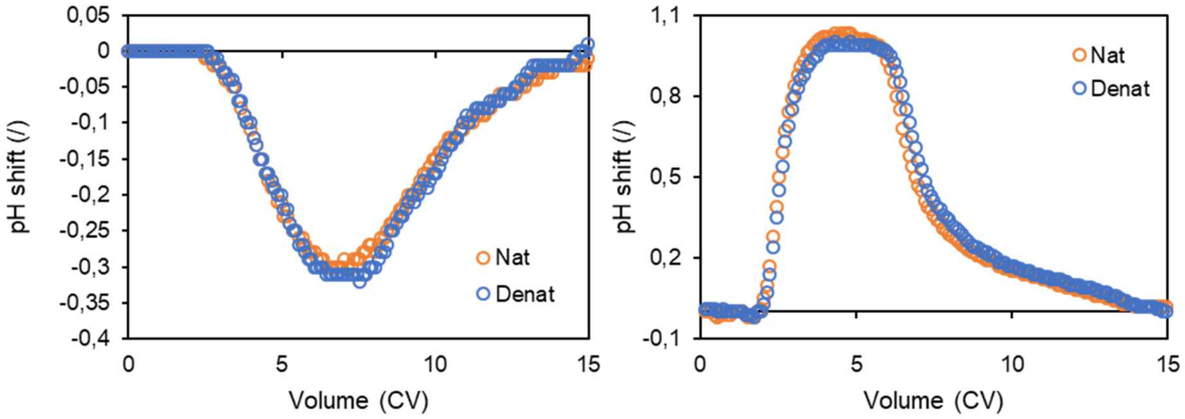

Figure S3. Effect of conformational changes of the protein pH profile. Stepwise buffer change from buffer A (1 mM lactate, pH 4.3) to buffer B (1 mM lactate, 1 M NaCl, pH 4.3) on the left and vice versa on the right. pH transitions with subtracted baseline of reference for the naturated (Nat) and denaturated (Denat) protein A.

4. Solution algorithm and limit of detection

A simple algorithm was developed that allows accurate experimental determination of the amount of immobilized protein and can be summarized as follows:

i) the pH transition method is applied to a blank matrix (without immobilized protein), as it may also have some ionic character;

ii) the blank matrix is immobilized with a selected protein and its amount is accurately determined by suitable non-destructive measurement (some of which are described in the Introduction section);

iii) the pH transition method is then applied to a selected buffer-immobilized protein system, and the blank response from step i) is subtracted from the obtained pH shift;

iv) the pH peak is evaluated based on its asymmetry - if the ratio between pH peak height and width, determined at 90% height, is below 4, a linear correlation between the pH peak height and the immobilized mass is expected. Otherwise, the buffer pH should be adjusted and steps i) and iii) repeated until the proposed criterion is met;

v) once the measurement falls within the linear range, the sensitivity and duration of the method can be adjusted by changing the buffer concentration (e.g., lowering the buffer concentration to increase sensitivity);

vi) when all of the above points are considered, the two-point linear correlation can be constructed using the measured point and the origin point (similar to Figure 2H). This correlation can then be used to determine the amount of immobilized protein.

This approach is similar to the two-point calibration commonly performed prior to measurements with any sensor. Although the mathematical model allows for in-silico

prediction of the pH transition, the potential effect of the matrix itself cannot be considered and should therefore be determined experimentally on a case-by-case basis. Therefore, this method allows a simple, direct and non-invasive determination of immobilized protein amount using biological buffers. Their concentration and pH affect the sensitivity and linear range of the method and are therefore a powerful tool to adjust conditions to a particular immobilized protein and its amount. While we are flexible with buffer adjustment to increase method sensitivity, there is lower limit of detection determined by exceedingly low buffer capacity required to detect extremely small quantities of immobilized protein, disabling method robustness and therefore reproducibility. Another challenge to the application of the method is that the immobilization matrix is charged since it significantly affects the shape of the pH transition profile. Although the pH transition profile of the original immobilization matrix (matrix prior to protein immobilization) is always measured and subtracted from the final pH transition signal, the accuracy of protein estimation is lower when the contribution of the matrix is comparable or even larger than that of the protein itself. The best accuracy of commercially available pH electrodes is in the range of 0.01-0.05 pH units[6]. Assuming a change of 0.05 pH units as the limit of quantification, one can determine a mass of about 50 µg of immobilized protein (corresponding to less than 2 nanomoles for protA), as demonstrated in Figure S4. The application of the proposed method is further facilitated by a developed mathematical model that accurately predicts the direction and magnitude of the pH shift. Its greatest strength is that it requires only protein amino acid sequence and buffer pK_a value(s). However, since many proteins can contain tags to facilitate their purification (e.g. 6xHis or GST)[7, 8], they should be also considered in a mathematical model calculation to correctly predict the pH transition profile. Still, proposed model is a powerful tool for in-silico design of pH measurements to adjust buffer conditions for required pH response and to establish conditions for a broad linear response range. Needless to say, due to its simplicity, all results can be obtained and analyzed

within a few minutes on a common PC, which is significantly faster compared to any laboratory experiment, not to mention the experimental costs.

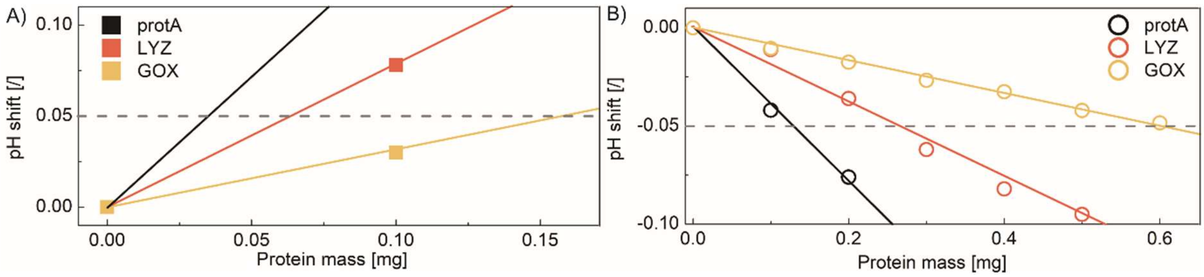

Figure S4. The amount of immobilized protein that can be detected accurately (identified as 0.05 pH unit shift) for the stepwise change from buffer A to buffer B (A) and buffer B to buffer A (B). Note that the zoomed Figure 4F was used to construct both figures in the desired area.

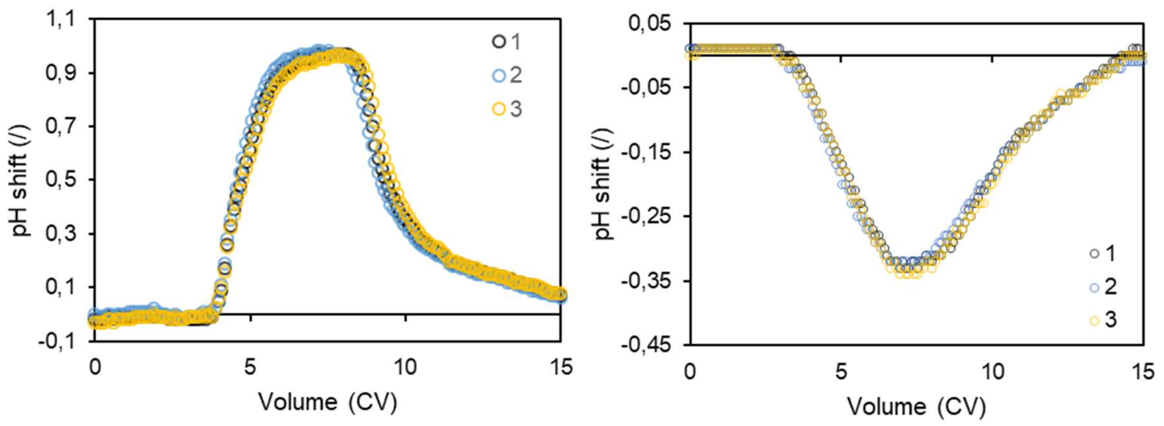

Figure S5. Robustness of the method. Stepwise buffer change from buffer A (1 mM lactate, pH 4.3) to buffer B (1 mM lactate, 1 M NaCl, pH 4.3) on the left and vice versa on the right. pH shifts with subtracted baseline of reference for repeated pH transition measurements for immobilized protein A.

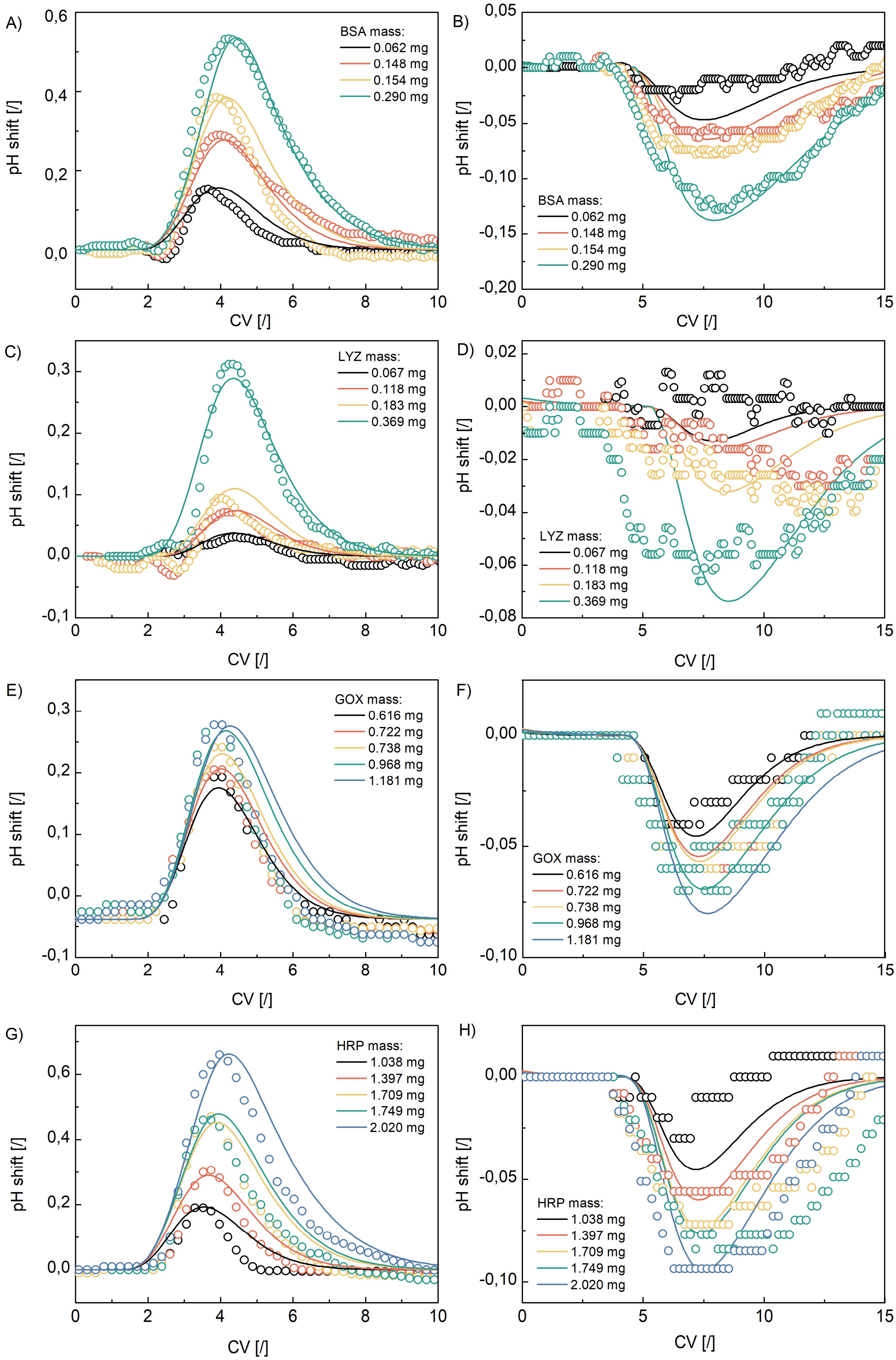
A)
B)
C)
D)
E)
F)
G)
H)
pH shift [/]
CV [/]
BSA mass:
0.062 mg
0.148 mg
0.154 mg
0.290 mg
LYZ mass:
0.067 mg
0.118 mg
0.183 mg
0.369 mg
GOX mass:
0.616 mg
0.722 mg
0.738 mg
0.968 mg
1.181 mg
HRP mass:
1.038 mg
1.397 mg
1.709 mg
1.749 mg
2.020 mg

Figure S6. Comparison of experimental-based (circles) and model-based (line) pH transition profiles for polyHIPE samples containing various amounts of immobilized proteins: BSA, LYZ, GOX and HRP. Buffer A: 1 mM lactate buffer, pH 4.3 (for BSA and LYZ) and 3.9 (for GOX and HRP); buffer B:1 mM lactate buffer with 1 M NaCl, pH 4.3 (for BSA and LYZ) and 3.9 (for GOX and HRP); flow rate 3 mL/min. The pH shift response for stepwise change from buffer A to buffer B (A, C, E, G) and buffer B to buffer A (B, D, F, H).

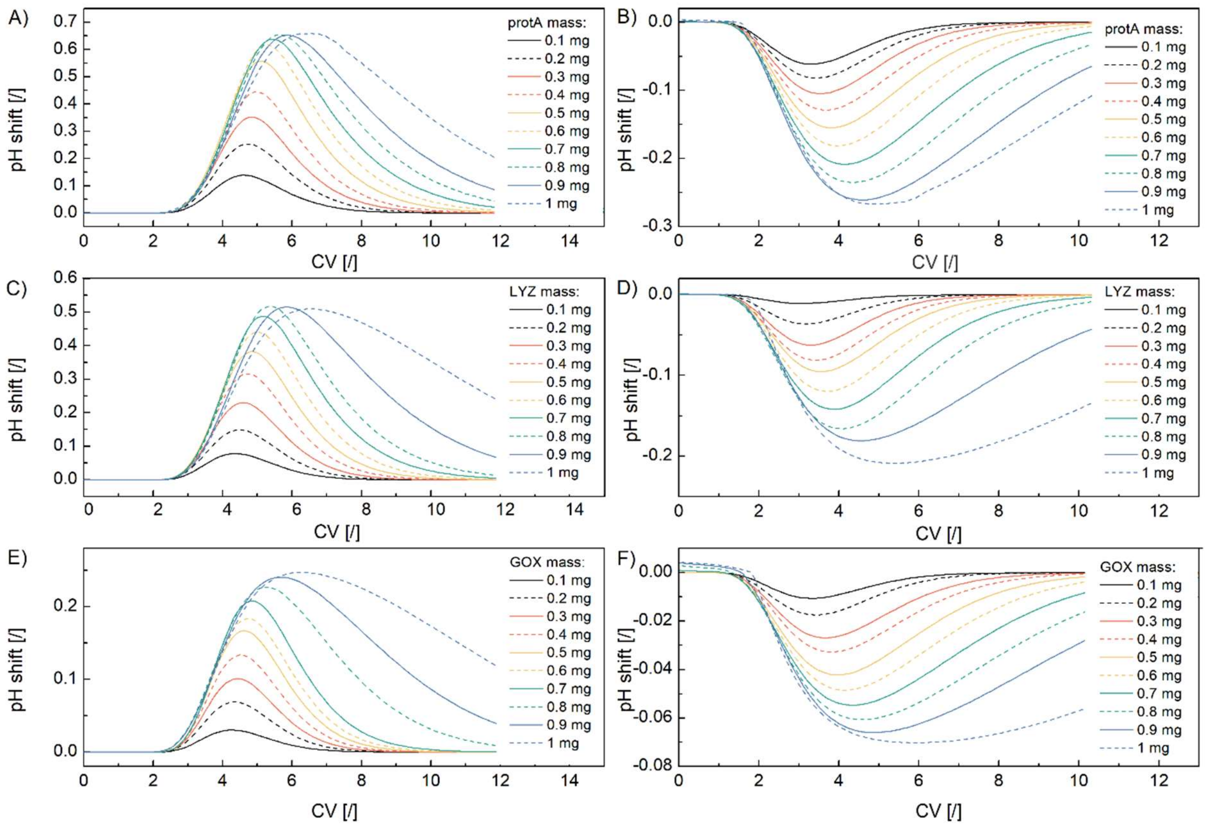

Figure S7. Model-based pH transition profiles for samples containing various masses of immobilized proteins (0.1 - 1.0 mg): protA (A-B), pH = 4.3; LYZ (C-D), pH = 4.3; and GOX (E-F), pH = 3.9: stepwise changes from buffer A to buffer B (A, C, E) and buffer B to buffer A (B, D, F) were performed.

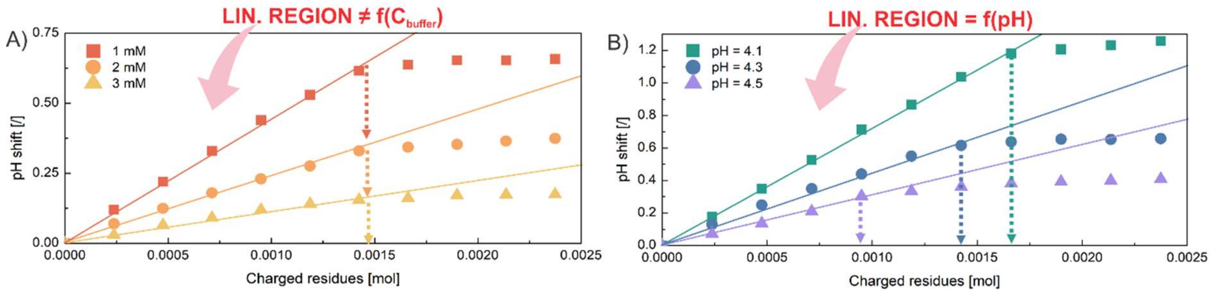

Figure S8. Model-based effect of buffer composition (concentration (A) and pH (B)) on the linear region of protA.

Table S1. Summary of immobilized protein properties

Charged residuals	pK$_a$*	Number of residues groups*				
		Protein A (28 kDa)	BSA (66 kDa)	LYZ (15 kDa)	GOX (160 kDa)	HRP (44 kDa)
aspartic acid	3.7	18	40	7	36	24
glutamic acid	4.3	24	59	2	30	8
histidine	6.1	3	17	1	20	6
cysteine	8.3	/	35	9	3	10
tyrosine	10.1	4	21	3	28	5
lysine	10.5	26	60	6	15	6
arginine	12.5	4	26	12	23	21

*based on primary amino acid sequence from ProtParm software.

Table S2. The pH peak asymmetry value determined at half peak height as a function of the immobilized mass of the protein: protA, LYZ and GOX

Mass [mg]	System					
	protA		LYZ		GOX	
	A to B	B to A	A to B	B to A	A to B	B to A
0.1	1.364	1.250	1.333	1.272	1.350	1.357
0.2	1.364	1.250	1.333	1.333	1.350	1.357
0.3	1.364	1.300	1.333	1.333	1.417	1.357
0.4	1.410	1.333	1.417	1.417	1.492	1.417
0.5	1.417	1.417	1.524	1.524	1.524	1.417
0.6	1.500	1.524	1.555	1.555	1.555	1.524
0.7	1.539	1.555	1.582	1.620	1.620	1.620
0.8	1.643	1.643	1.643	1.833	1.944	1.833
0.9	1.867	1.833	1.941	2.125	2.054	2.090
1.0	2.346	2.136	2.125	2.370	2.250	2.270

Table S3. The peak height vs peak width (determined at 90% of peak height) ratio (H/W) for all experimentally-determined pH transition for stepwise change from buffer A to buffer B. For comparison the pH peak asymmetry determined at half peak height based on model predicted pH peaks are also listed. Note that colored values represent systems that fall outside the linear correlation between the height of the pH transition peak and the immobilized mass (identified as values above 4.0 for H/W ratio and values below 2.0 for peak asymmetry)

Mass [mg]	H/W	Asymmetry	Mass	H/W	Asymmetry
	protA			BSA	
0.25	3.1	1.36	0.062	3.67	1.40
0.42	3.1	1.36	0.148	3.14	1.40
0.51	3.0	1.46	0.154	2.08	1.40
0.72	3.1	1.56	0.290	2.06	1.50
0.88	3.1	1.67		LYZ	
0.98	5.5	2.11	0.067	/	1.40
			0.118	/	1.44
Buffer conc. [mM]	protA		0.183	3.31	1.44
0.25	4.31	2.00	0.369	2.90	1.50
0.50	3.90	2.00		GOX	
1.00	1.10	1.36	0.616	3.89	1.18
2.00	1.08	1.33	0.722	3.75	1.40
			0.738	2.83	1.40
Buffer pH [/]	protA		0.968	2.69	1.43
3.9	0.99	1.17	1.181	3.16	1.45
4.1	1.50	1.17		HRP	
4.3	1.83	1.38	1.038	3.16	1.40
4.5	4.81	2.00	1.397	2.12	1.40
4.7	5.00	2.27	1.709	1.70	1.45
			1.749	1.96	1.49
			2.020	1.44	1.55

References

1. T. M. Pabst, G. Carta, N. Ramasubramanyan, A. K. Hunter, P. Mensah and M. E. Gustafson, *Biotechnology Progress*, 2008, **24**, 1096-1106.
2. T. A. Vetter, G. Ferreira, D. Robbins and G. Carta, *Separation Science and Technology*, 2014, **49**, 477-489.
3. T. M. Pabst, D. Antos, G. Carta, N. Ramasubramanyan and A. K. Hunter, *Journal of Chromatography A*, 2008, **1181**, 83-94.
4. T. M. Pabst and G. Carta, *Journal of Chromatography A*, 2007, **1142**, 19-31.
5. B. J. Bennion and V. Daggett, *Proceedings of the National Academy of Sciences of the United States of America*, 2003, **100**, 5142-5147.
6. S. E. Mowbray and A. M. Amiri, *Diagnostics*, 2019, **9**.
7. V. Mishra, *Current Protein & Peptide Science*, 2020, **21**, 821-830.
8. M. Linhult, S. Gulich and S. Hober, *Protein and Peptide Letters*, 2005, **12**, 305-310.
